# Clinical Features and Risk Factors for In-Hospital Mortality From COVID-19 Infection at a Tertiary Care Medical Center, at the Onset of the US COVID-19 Pandemic

**DOI:** 10.1177/08850666211001799

**Published:** 2021-03-24

**Authors:** Ashish Bhargava, Susanna M. Szpunar, Mamta Sharma, Elisa Akagi Fukushima, Sami Hoshi, Miriam Levine, Nikhil Gandhi, Wei Zhao, Somero Michael, Farah Tanveer, Dima Youssef, Meredith Coyle, Johnson Leonard, Louis Saravolatz

**Affiliations:** 1 Division of Infectious Diseases, Department of Internal Medicine, Thomas Mackey Center for Infectious Disease Research, 21928 Ascension St. John Hospital, Detroit, MI, USA

**Keywords:** hospital mortality, infections, clinical research

## Abstract

**Background:**

Mortality from COVID-19 has been associated with older age, black race, and comorbidities including obesity, Understanding the clinical risk factors and laboratory biomarkers associated with severe and fatal COVID-19 will allow early interventions to help mitigate adverse outcomes. Our study identified risk factors for in-hospital mortality among patients with COVID-19 infection at a tertiary care center, in Detroit, Michigan.

**Methods:**

We conducted a single-center, retrospective cohort study at a 776-bed tertiary care urban academic medical center. Adult inpatients with confirmed COVID-19 (nasopharyngeal swab testing positive by real-time reverse-transcriptase-polymerase-chain-reaction (RT-PCR) assay) from March 8, 2020, to June 14, 2020, were included. Clinical information including the presence of comorbid conditions (according to the Charlson Weighted Index of Comorbidity (CWIC)), initial vital signs, admission laboratory markers and management data were collected. The primary outcome was in-hospital mortality.

**Results:**

Among 565 hospitalized patients, 172 patients died for a case fatality rate of 30.4%. The mean (SD) age of the cohort was 64.4 (16.2) years, and 294 (52.0%) were male. The patients who died were significantly older (mean [SD] age, 70.4 [14.1] years vs 61.7 [16.1] years; *P* < 0.0001), more likely to have congestive heart failure (35 [20.3%] vs 47 [12.0%]; *P* = 0.009), dementia (47 [27.3%] vs 48 [12.2%]; *P* < 0.0001), hemiplegia (18 [10.5%] vs 18 [4.8%]; *P* = 0.01) and a diagnosis of malignancy (16 [9.3%] vs 18 [4.6%]; *P* = 0.03).From multivariable analysis, factors associated with an increased odds of death were age greater than 60 years (OR = 2.2, *P* = 0.003), CWIC score (OR = 1.1, *P* = 0.023), qSOFA (OR = 1.7, *P* < 0.0001), WBC counts *(*OR = 1.1*, P* = 0.002), lymphocytopenia (OR = 2.0, *P* = 0.003), thrombocytopenia (OR = 1.9, *P* = 0.019), albumin *(*OR = 0.6*, P* = 0.014), and AST levels *(*OR = 2.0, *P* = 0.004) on admission.

**Conclusions:**

This study identified risk factor for in-hospital mortality among patients admitted with COVID-19 in a tertiary care hospital at the onset of U.S. Covid-19 pandemic. After adjusting for age, CWIC score, and laboratory data, qSOFA remained an independent predictor of mortality. Knowing these risk factors may help identify patients who would benefit from close observations and early interventions.

Severe acute respiratory syndrome coronavirus 2 (SARS-CoV-2), is a novel coronavirus that causes coronavirus disease 2019 (COVID-19).^1,2^ It was identified in December 2019 in Wuhan, China and has become pandemic.^3^ Worldwide, 78,145,043 confirmed COVID-19 cases with 1,719,973 deaths were reported as of December 23, 2020.^4^ In the United States, a total of 18,238,314 cases have been reported with 322,851 deaths. Michigan had the highest observed case-fatality rate (CFR) in the United States in the early phase of U.S. pandemic.^4,5^

Older age, black race and comorbidities including obesity are suggested risk factors for death from COVID-19. Studies from multiple countries revealed association between age >65 years and comorbidities and increased risk of death.^6–8^ Preliminary U.S. data showed that 80% of deaths associated with COVID-19 were among older adults.^9^ Persons with diabetes and cardiovascular disease also had higher COVID-19-associated mortality.^10^ A study also reported a high death rate among nursing facilities, as expected from older and chronically ill populations.^11^ The Centers for Disease Control and Preventions (CDC) aligns underlying causes of health disparities that include social determinants of health, racism and discrimination, economic and educational disadvantages, health care access and quality, and occupation. Miller et al reported significantly higher rates of COVID-19 diagnosis and death in disproportionately black counties which also had a greater incidence of diabetes, heart disease deaths and cerebrovascular deaths.^12^ Black/African-Americans comprise only 14% of Michigan’s population, they accounted for 40% of deaths due to COVID-19.^13^ Obesity is often associated with chronic conditions, such as diabetes, hypertension, cardiac conditions, and cerebrovascular disease.

In a study from an integrated health system patient with a BMI of 18.5 to 24 kg/m^2^ in comparison to those with BMIs of 40 to 44 kg/m^2^ and greater than 45 kg/m^2^ the relative risk of dying was 2.68 (95% CI, 1.43-5.04) and 4.18 (CI, 2.12-8.26), respectively. Increased risk for pro-inflammatory and prothrombotic states as well as poor ventilatory lung mechanics with obesity are poor prognostic factors.^14^ Our study identified risk factors at the time of admission for in-hospital mortality among COVID-19 patients in the early phase of this pandemic in a tertiary care hospital located in Detroit, Michigan.

## Study Setting and Design

We conducted a single-center, retrospective cohort study at a 776-bed tertiary care urban academic medical center. The study was approved by the Ascension St John Hospital Institutional Review Board. Adult patients with confirmed COVID-19 (positive real-time reverse-transcriptase-polymerase-chain-reaction (RT-PCR) assay of a nasopharyngeal swab) from March 8, 2020, to June 14, 2020, were included.

## Data Collection

Data were collected from the electronic medical record for all patients meeting inclusion criteria. Demographics included age, gender, race, and residential 9-digit zip code. Clinical information included the presence of comorbid conditions (according to the Charlson Weighted Index of Comorbidity), home medications, presenting symptoms, initial vital signs, admission laboratory and radiological findings, and outcome variables including the discharge disposition.

## Definitions

Age was categorized both as quartiles and as ≥60 vs <60 years. Obesity and morbid obesity were defined according to CDC definitions.^15^ Pre-existing renal disease was defined as chronic dialysis, history of renal transplant, uremic syndrome, or a creatinine >3 mg/dL on prior admissions. Malignancy was included if active or treated in the last 5 years. Fever was defined as an oral temperature of 37.8 °C or higher. Leukopenia and lymphocytopenia were defined as white blood cell counts of less than 4 × 10^9^/L and absolute lymphocyte count less than 1.0 × 10^9^/L, respectively. Thrombocytopenia was defined as a platelet count less than 150 × 10^9^/L. Elevated ferritin and d dimer were defined as 4 and 6 times the upper limit of normal, respectively. Acute renal injury was defined as an increase in serum creatinine by ≥0.3 mg/dL (≥26.5 µmol/L) within 48 hours or an increase in serum creatinine to ≥1.5 times baseline, known or presumed to have occurred within the prior 7 days.^16^ COVID-19 pneumonia was defined as an acute respiratory disorder meeting at least 3 out of 4 criteria: respiratory signs/symptoms (cough/dyspnea/tachypnea), fever, oxygen saturation below 94%, and abnormal chest x-ray at the time of hospital admission. Uncomplicated illness, severe pneumonia, acute respiratory distress syndrome (ARDS) and septic shock were defined according to the WHO definitions.^17^ The 5-digit ZIP code was also used to collect median income from 2017 United States census data.^18^

## Statistical Analysis

Statistical analysis was performed using SPSS v. 27.0 (Armonk, NY). Descriptive statistics were generated to characterize the study group. Continuous variables were described as the mean with standard deviation or median with interquartile range; categorical variables were described as frequency distributions. Univariable analysis was done using Student’s t-test, the Mann-Whitney U test and chi-squared analysis. Variables that were found to be significant or near-significant (*P* < 0.09) predictors of mortality were then entered a multivariable logistic regression model using a forward likelihood ratio algorithm. When 2 variables were measuring the same underlying factor, the variable with the highest univariable measure of association was used in the model. Results from the regression are reported as odds ratios with 95% confidence intervals. All reported *P* values are 2-sided.

## Results

A total of 565 hospitalized patients with confirmed SARS-CoV-2 infection were included. The mean (Standard Deviation—[SD]) age of the cohort was 64.4 (16.2) years, 294 (52.0%) were male and 436 (77.2%) were black/African-American. The mean body mass index (BMI) of the cohort was 32.0 (9.02) kg/m^2^. At least one comorbidity was present in 538 (95.2%) of patients. The most common co-morbidities were hypertension (418, 74.0%), obesity (300, 53.1%), and diabetes (219, 38.8%). The mean duration of symptoms prior to hospitalization was 5.29 (4.1) days. Fever was noted in 212 (37.5%) patients. An abnormal admission chest x-ray was found in 423 (74.9%) patients, and pneumonia was diagnosed in 302 (53.5%) patients. ICU care was required for 140 (24.8%) patients and mechanical ventilation for 135 (23.9%). Overall, 393 (69.6%) improved clinically and survived to discharge.

The in-hospital CFR was 30.4% (172/565). Patients who died were older (mean [SD] age, 70.4 [14.0] years vs 61.8 [16.4] years; *P* < 0.0001) than patients who survived. The chi-squared test for trend showed a linear trend of increasing mortality by increase in age group. Males were more likely to die (99 [57.6%] vs 73 [42.4%]; *P* = 0.08) than females. There was no association between race and mortality (*P* = 0.19) (Table 1).

**Table 1.** Univariable Analysis of Predictors for Death From COVID-19 Infection.

Characteristic	Survivors (n = 393) (%)	Died (n = 172) (%)	OR (95% CI)	*P* value
**Age groups, years**				
<60	178 (45.3)	36 (20.9)	3.1 (2.1, 4.7)	<0.0001
≥60	215 (54.7)	136 (79.1)		
**Age quartiles, years, *n* (%)**				
<53	117 (29.8)	19 (11.0)		<0.0001
53-65.9	105 (26.7)	38 (22.1)		
66-74.9	83 (21.1)	47 (27.3)		
≥75	88 (22.4)	68 (39.5)		
**Sex, *n* (%)**				
Male	195 (49.6)	99 (57.6)	1.4 (1.0, 2.0)	0.08
Female	198 (50.4)	73 (42.4)		
**Race, *n* (%)**				
White	73 (18.7)	42 (24.4)		0.19
Black	308 (78.8)	128 (74.4)		
Other	10 (2.6)	2 (1.2)		
**BMI, mean ± SD**	32.4 ± 8.5	31.1 ± 10.1		0.14
**Median income, mean ± SD**	36477.1 ± 14926.7	39131.9 ± 17013.2		0.07
**Admission source**				
Home	297 (75.6)	99 (57.6)	2.3 (1.6, 3.3)	<0.0001
Nursing facility	96 (24.4)	73 (42.4)		
**Comorbidities, *n* (%)**				
≥one comorbidity	370 (94.1)	168 (97.7)	2.6 (0.9, 7.7)	0.07
Myocardial infarction	28 (7.1)	13 (7.6)	1.1 (0.5, 2.1)	0.86
Congestive heart failure	47 (12.0)	35 (20.3)	1.9 (1.2, 3.0)	0.009
Peripheral vascular disease	27 (6.9)	11 (6.4)	0.9 (0.5, 1.9)	0.84
Cerebrovascular disease	61 (15.6)	25 (14.5)	0.9 (0.6, 1.5)	0.76
Dementia	48 (12.2)	47 (27.3)	2.7 (1.7, 4.2)	<0.0001
Chronic pulmonary disease	83 (21.9)	43 (25.0)	1.2 (0.8, 1.8)	0.42
Connective tissue disease	6 (1.5)	5 (2.9)	1.9 (0.6, 6.4)	0.28
Peptic ulcer disease	12 (3.1)	6 (3.5)	1.1 (0.4, 3.1)	0.79
Diabetes	148 (37.7)	71 (41.3)	1.2 (0.8, 1.7)	0.42
Hemiplegia	18 (4.8)	18 (10.5)	2.3 (1.2, 4.5)	0.013
Renal disease	64 (16.3)	36 (20.9)	1.4 (0.9, 2.1)	0.19
Any malignancy	18 (4.6)	16 (9.3)	2.1 (1.1, 4.3)	0.03
Metastatic solid tumor	7 (1.8)	5 (2.9)	1.7 (0.5, 5.3)	0.39
Mild liver disease	7 (1.8)	4 (2.3)	1.3 (0.4, 4.5)	0.67
Moderate-severe liver disease	1 (0.3)	4 (2.3)	9.3 (1.0, 84.1)	0.02
AIDS	3 (0.8)	0 (0.0)	—	—
Median CWIC (25th, 75th)	1 (0,2)	2 (1,3)		<0.0001
Hypertension	282 (71.8)	136 (79.1)	1.5 (1.0, 2.3)	0.07
Current tobacco smoker	25 (6.4)	12 (7.1)	1.1 (0.5, 2.3)	0.76
Obesity	216 (56.0)	84 (49.1)	0.8 (0.5, 1.1)	0.14
Morbid obesity	75 (19.4)	30 (17.5)	0.9 (0.6, 1.4)	0.60
**Symptoms, *n* (%)**				
Fever	227 (58.4)	87 (51.2)	0.7 (0.5, 1.1)	0.12
Shortness of breath	271 (69.7)	129 (76.3)	1.4 (0.9, 2.1)	0.11
Altered mental status	83 (21.1)	76 (44.2)	3.0 (2.0, 4.4)	<0.0001
**Vitals on admission**				
Systolic BP, mean ± SD	133.9 ± 24.8	132.7 ± 28.0		0.65
Systolic BP on admission				
>100 mmHg	365 (92.9)	147 (85.5)	2.2 (1.3, 3.9)	0.005
<100 mmHg	28 (7.1)	25 (14.5)		
Diastolic BP, mean ± SD	75.0 ± 17.4	73.6 ± 18.1		0.36
Heart rate, mean ± SD	99.0 ± 21.7	104.6 ± 22.9		0.006
Respiratory rate on admission				
<22 breaths per minute	217 (55.2)	59 (34.3)	2.4 (1.6, 3.4)	<0.0001
≥22 breaths per minute	176 (44.8)	113 (65.7)		
Fever, *n* (%)	137 (34.9)	75 (43.6)	1.5 (1.0, 2.1)	0.05
Oxygen saturation, mean ± SD	0.95 ± 0.06	0.92 ± 0.08		<0.0001
Mean qSOFA score	0.73 ± 0.69	1.24 ± 0.81		<0.0001
**Abnormal chest x-ray on admission, *n* (%)**	283 (72.2)	140 (81.4)	1.7 (1.1, 2.6)	0.02
**Laboratory findings on admission, *n* (%)**				
Leukopenia	44 (11.2)	16 (9.3)	0.8 (0.4, 1.5)	0.50
Lymphocytopenia	172 (47.0)	104 (61.2)	1.9 (1.3, 2.7)	0.001
Thrombocytopenia	67 (17.2)	50 (29.2)	2.0 (1.3, 3.0)	0.001
Elevated AST (>40 U/L)	191 (51.8)	108 (67.5)	1.9 (1.3, 2.9)	0.001
Elevated ALT (>40 U/L)	130 (34.3)	46 (27.7)	0.7 (0.5, 1.1)	0.13
Low serum proteins (<6.2 gm/dl)	26 (6.8)	23 (13.9)	2.2 (1.2, 4.0)	0.004
Low serum albumin (<3.5 gm/dl)	148 (38.8)	110 (66.3)	3.1 (2.1, 4.5)	<0.0001
Elevated serum LDH (>500 U/L)	46 (19.6)	38 (38.8)	2.6 (1.5, 4.3)	<0.0001
Elevated serum ferritin (>1000 ng/ml)	90 (35.9)	52 (45.6)	1.5 (1.0, 2.4)	0.08
Elevated D dimer (>1500 ng/ml)	73 (50.3)	51 (75.7)	3.1 (1.6, 5.7)	<0.0001
Elevated troponin (>0.05 ng/ml)	50 (18.1)	43 (38.1)	2.9 (1.7, 4.5)	<0.0001
Elevated CRP (>10 mg/L)	332 (91.0)	140 (95.3)	2.1 (0.9, 4.9)	0.07
Elevated procalcitonin (>0.10 ng/dl)	231 (70.2)	135 (88.8)	3.4 (1.9, 5.9)	<0.0001
Elevated creatinine (from baseline)	134 (35.3)	88 (55.3)	2.3 (1.6, 3.3)	<0.0001
**Clinical diagnosis, *n* (%)**				
Uncomplicated COVID19 illness	203 (51.7)	60 (34.9)		<0.0001
Mild COVID-19 infection	136 (34.6)	61 (35.5)		
Severe COVID-19 infection	54 (13.7)	51 (29.7)		

Abbreviations: *n,* number; OR, odds ratio; CI, confidence interval; BMI, body mass index; SD, standard deviation; AIDS, acquired immunodeficiency syndrome; CWIC, Charlson weighted index of comorbidity; BP, blood pressure; ACEI, angiotensin converting enzyme inhibitor; ARBs, angiotensin II receptor blockers; AST, aspartate aminotransferase; ALT, alanine aminotransferase; LDH, lactate dehydrogenase; CRP, C-reactive protein; qSOFA, quick sepsis related organ failure assessment.

Patients who died were more likely to have congestive heart failure (20.3% vs 12.0%; *P* = 0.009), dementia (27.3% vs 12.2%; *P* < 0.0001), hemiplegia (10.5% vs 4.8%; *P* = 0.01) and a diagnosis of malignancy (9.3% vs 4.6%; *P* = 0.03). The mean duration of symptoms prior to the hospitalization was shorter for patients who died compared to those survived (4.8 ± 4.1 vs 5.5 ± 4.1; *P* = 0.05). Among patients who died, 44.2% were confused on presentation compared to 21.1% of those who survived (*P* < 0.0001). Mean qSOFA score were significantly higher among patients who died compared to those who survived.

Patients who died from COVID-19 infection demonstrated an increased inflammatory response, including higher mean white blood cell counts (WBCs) (× 10^9^ per L [SD] 8.6 [4.3] vs 7.5 [3.8]; *P* = 0.005), lower absolute lymphocyte counts (× 10^9^ per L [SD] 1.0 [0.7] vs 1.2 [0.6]; *P* = 0.003), and increased C-reactive protein (CRP) levels (mean [SD] 130.0 [92.4] vs 88.1 [73.3]; *P* < 0.0001) compared with patients who survived. Patients who died had significantly elevated procalcitonin and troponin levels on admission; however, these patients also had acute renal injury on admission which may explain these findings.

Patients who died were intubated sooner (Median [IQR] 22.3 [101.3] vs 45.2 [69.5] hours; *P* = 0.8) than who survived. Use of azithromycin, hydroxychloroquine and steroids was significantly higher among patients who died than survived. The CFR among patients requiring intubation and patients admitted to ICU was 77.0% and 75.7%, respectively (Table 2).

**Table 2.** Outcome of COVID-19 Infected Patients.

Complications, n (%)	Survivors (n = 393) (%)	Died (n = 172) (%)	OR (95% CI)	*P* value
Mechanical intubation	31 (7.9)	104 (60.5)	17.9 (11.1, 28.8)	<0.0001
Time to intubation				
<24 hrs. from hospital admission	14 (45.2)	55 (52.9)	0.7 (0.3, 1.6)	0.45
>24 hrs. from hospital admission	17 (54.8)	47 (47.1)		
ICU admission	34 (8.7)	106 (61.6)	17.0 (10.6, 27.1)	<0.0001
Septic shock	8 (2.0)	64 (37.2)	28.4 (13.2, 61.1)	<0.0001
ARDS	11 (2.8)	41 (23.8)	10.9 (5.4, 21.8)	<0.0001
Acute renal injury	120 (30.5)	114 (66.3)	4.5 (3.1, 6.6)	<0.0001
DIC	1 (0.3)	3 (1.7)	6.9 (0.7, 67.4)	0.05
Rhabdomyolysis	12 (3.1)	7 (4.1)	1.3 (0.5, 3.5)	0.55
Need for RRT	6 (1.5)	18 (10.6)	7.6 (3.0, 19.6)	<0.0001
Azithromycin given	214 (54.6)	115 (67.3)	1.7 (1.2, 2.5)	0.005
Hydroxychloroquine	223 (56.7)	133 (77.3)	2.6 (1.7, 3.9)	<0.0001
Steroids	158 (40.2)	125 (72.7)	4.0 (2.7, 5.9)	<0.0001

Abbreviations: *n*, number; OR, odds ratio; CI, confidence interval; ICU, intensive care unit; ARDS, adult respiratory distress syndrome; DIC, disseminated intravascular coagulation; RRT, renal replacement therapies.

From the univariable analysis in Table 1, we identified each variable that showed and/or reached statistical significance with *P* < 0.09 between patients who were discharged versus those who died. The CWIC score was used in the model instead of separate comorbidities. For multivariable logistic regression, variables initially entered the model included age ≥60 yrs., sex, CWIC, median income, hospital admission source, hypertension, qSOFA score, WBC counts on admission, lymphocytopenia on admission, thrombocytopenia on admission, creatinine on admission, and aspartate aminotransferase (AST) on admission, total protein on admission, albumin on admission, and abnormal chest x-ray on admission. After 8 iterations, the model with the lowest -2 log likelihood value included 8 variables that were associated with the odds of death from COVID-19 infection, including patient age (≥60 yrs.), higher CWIC score, higher qSOFA score, higher WBC counts on admission, lower albumin on admission, elevated AST, lymphocytopenia, and thrombocytopenia on admission (Table 3).

**Table 3.** Multivariable Analysis of Predictors for Death From COVID-19 Infection.

Variables	OR (95% CI)	*P* value
Age ≥ 60 yrs.	2.2 (1.3, 3.7)	0.003
CWIC at hospital admission	1.1 (1.0, 1.3)	0.023
qSOFA	1.7 (1.3, 2.3)	<0.0001
WBC on admission	1.1 (1.0, 1.2)	0.002
Lymphocytopenia on hospital admission	2.0 (1.2, 3.1)	0.003
Thrombocytopenia on hospital admission	1.9 (1.1, 3.2)	0.019
Albumin levels on hospital admission	0.6 (0.4, 0.9)	0.014
AST >40	2.0 (1.2, 3.1)	0.004

Abbreviations: OR, odds ratio; CI, confidence interval; CWIC, Charlson weighted index of comorbidity; qSOFA, quick sepsis related organ failure assessment; WBC, white blood cells; AST, aspartate aminotransferase.

## Discussion

In our study, the CFR was 30.4%, similar to the 30.9% CFR reported from Georgia (U.S.).^19^ Our institution noted an early surge of COVID-19 cases like New York, when ventilatory and other therapeutic interventions were evolving.^20^ Nearly 40% of studied patients were transitioned to comfort care, and the predominantly older cohort also may explain the high mortality seen in our study. In addition, our population included 30% of patients from nursing facilities who had worse prognosis. Lower mortality rates have been reported in early studies from different countries but recent data from various US cohorts demonstrate a mortality range from 21% to 67%.^6,7,9,10,19–25^ Risk factors for death in our study generally were consistent with prior data other than obesity and race which were not associated with mortality.

Age was also an independent risk factor for death in our study, which confirms prior reports. An early study from China reported higher mortality in older age groups.^6^ Another study also found significantly higher mortality in patients ≥64 years old compared with younger patients (36% vs 15%; *P* < .001).^26^ Early U.S. epidemiologic data suggest that the case fatality is highest in persons aged ≥85 years (range 10%-27%), followed by 3% to 11% for ages 65 to 84 years.^11^ Poorer outcomes in the elderly may result from immunosenescence and immune dysregulation leading to insufficient immunologic or overexuberant inflammatory responses.

Similar to the findings of Eboni.et.al, a higher CWIC score was an independent predictor of mortality in our study.^22^ In particular, congestive heart failure, dementia, hemiplegia, malignancy, and severe liver disease were associated with mortality. Chinese studies have also previously reported high mortality in patients with comorbidities (10.5% for cardiovascular disease, 7.3% for diabetes, 6.3% for chronic respiratory disease, 6% for hypertension, and 5.6% for cancer).^6^ Another study reported a 28% CFR overall among patients with malignancies, 37% among hematological malignancies, and 25% among solid organ malignancies.^27^ The effect of multiple comorbidities (≥2) was synergistic, leading to a mortality of 15.4% compared to 5.6% in patients with one comorbidity.^28^

In our study, the qSOFA at the time of hospitalization was an independent predictor for in-hospital mortality from COVID-19. The qSOFA score was introduced by Third International Consensus Definitions for Sepsis and Septic Shock (Sepsis-3) task force and its use was associated with a greater prognostic accuracy for in-hospital mortality compared to either systemic inflammatory response syndrome (SIRS) or severe sepsis.^29^ We previously showed that bedside qSOFA score at the time of hospitalization can predict in-hospital mortality among adults ≤65 years with COVID-19 after adjusting for CWIC score and thrombocytopenia.^30^ In that study, for patients with qSOFA score of 0, 1, 2 and 3 at the time of hospital admission, the mortality rates were 8.7%, 19.7%, 41.7% and 60%, respectively.^30^ An early study from China noted higher SOFA scores on admission were associated with a higher odds of in-hospital death.^20^ There is limited information on qSOFA as a predictor of mortality. We found admission qSOFA to be an independent predictor for in-hospital mortality from COVID-19 regardless of age. Elevated respiratory rate, low oxygen saturation and need for oxygenation at the time of hospitalization have been associated with severe diseases and poor outcomes.^31,32^

Our study showed an inverse relationship between the level of albumin and the risk of death in COVID-19 patients. This result is consistent with a previous study reporting hypoalbuminemia associated with the COVID-19-related complications including death.^33,34^ It had been noted that change in albumin does not parallel the severity of hepatocellular injury in COVID-19, suggesting that there are other mechanisms involved for hypoalbuminemia in severe COVID-19.^35^ In a recently published article, the low albumin was considered as a predictor of mortality in COVID-19. A possible additional explanation was that the oxidative stress impairs the antioxidant property of the albumin and elicits cell and tissue damage.^36^

A research study conducted in China, assessed the association between markers of liver injury and mortality in COVID-19 patients.^37^ It was found that AST elevation upon hospital admission was positively associated with the increase of neutrophil counts and the decrease of lymphocyte counts at baseline. We now know that both neutrophilia and lymphopenia are indicators of disease severity. In our study, AST was associated with increased mortality, similar finding with European study where liver enzyme elevations were associated with mortality and ICU admission among COVID-19 patients.^38^

We also found that WBC counts, lymphocytopenia, and thrombocytopenia at the time of hospital admission were independent predictors for in-hospital mortality. In our cohort, lymphocytopenia was more frequent in patients who died versus those who survived (62.5% vs 46.9%). A systematic review and meta-analysis showed that patients who died had lower lymphocyte count (mean difference − 395.35 µL [− 165.64, − 625.07], *P* < 0.001).^39^ Thrombocytopenia has been linked with severe COVID-19 complications including death.^32,40^ Non-survivors have been reported to have higher WBC counts than survivors, which is thought to be compensatory for a drop in other bloodlines.^35^

Our study has several limitations. This was a single institution study of admitted patients which makes generalization difficult. Because of the retrospective design, certain laboratory results were sometimes unavailable for some patients, including lactate dehydrogenase, d dimer, and serum ferritin. Patients with chronic lung disease and conditions associated with immunosuppression were only a small percentage among hospitalized patients. Therefore, the role of some of these variables in predicting mortality from COVID-19 infection could have been underestimated. Finally, a predominantly black and obese cohort may limit the generalizability of some findings (for e.g.: race). Nonetheless, our study involved a population of black patients in Detroit MI, a state that had the highest case-fatality rate in the United States at an early phase of pandemic. These data may provide valuable information on the risk factors for mortality in this population.

## Conclusions

Our study showed a mortality of 30.4% among hospitalized patients due to COVID-19 infection. Independent factors associated with in-hospital mortality were age ≥60 years, CWIC score, qSOFA score at the time of hospitalization, admission WBC, serum albumin, AST, lymphocytopenia, and thrombocytopenia at the time of admission. Recognizing the population at highest risk for severe COVID-19 related outcomes may guide and result in aggressive supportive care and treatment interventions.
